# Getting to zero the biomedical way in Africa: outcomes of deliberation at the 2013 Biomedical HIV Prevention Forum in Abuja, Nigeria

**DOI:** 10.1186/1753-6561-8-S3-S1

**Published:** 2014-09-11

**Authors:** Morenike Oluwatoyin Folayan, Megan Gottemoeller, Rosemary Mburu, Brandon Brown

**Affiliations:** 1 Department of Child Dental Health, Obafemi Awolowo University, Ile-Ife, Nigeria; 2 New HIV Vaccine and Microbicide Advocacy Society, Lagos, Nigeria; 3 Institute of Public Health, Obafemi Awolowo University, Ile-Ife, Nigeria; 4 Salter>Mitchell Consultancy, USA; 5 World AIDS Campaign, South Africa; 6 Africa Civil Society Platform on Health, South Africa; 7 University of California, Irvine, CA, USA

**Keywords:** Biomedical, HIV, prevention, Forum, meeting, report

## Abstract

**Background:**

Over the last few decades, biomedical HIV prevention research had engaged multiple African stakeholders. There have however been few platforms to enable regional stakeholders to engage with one another. In partnership with the World AIDS Campaign International, the Institute of Public Health of Obafemi Awolowo University, and the National Agency for the Control of AIDS in Nigeria, the New HIV Vaccine and Microbicide Advocacy Society hosted a forum on biomedical HIV prevention research in Africa. Stakeholders’ present explored evidences related to biomedical HIV prevention research and development in Africa, and made recommendations to inform policy, guidelines and future research agenda.

**Discussion:**

The BHPF hosted 342 participants. Topics discussed included the use of antiretrovirals for HIV prevention, considerations for biomedical HIV prevention among key populations; HIV vaccine development; HIV cure; community and civil society engagement; and ethical considerations in implementation of biomedical HIV prevention research. Participants identified challenges for implementation of proven efficacious interventions and discovery of other new prevention options for Africa. Concerns raised included limited funding by African governments, lack of cohesive advocacy and policy agenda for biomedical HIV prevention research and development by Africa, varied ethical practices, and limited support to communities’ capacity to actively engaged with clinical trial conducts. Participants recommended that the African Government implement the Abuja +12 declaration; the civil society build stronger partnerships with diverse stakeholders, and develop a coherent advocacy agenda that also enhances community research literacy; and researchers and sponsors of trials on the African continent establish a process for determining appropriate standards for trial conduct on the continent.

**Conclusion:**

By highlighting key considerations for biomedical HIV prevention research and development in Africa, the forum has helped identify key advocacy issues that Civil Society can expend efforts on so as to strengthen support for future biomedical HIV prevention research on the continent.

## Introduction

The New HIV Vaccine and Microbicide Advocacy Society and the World AIDS Campaign International in collaboration with the Institute of Public Health, Obafemi Awolowo University, Ile-Ife Nigeria and the National Agency for the Control of AIDS, Nigeria convened the 2013 Biomedical HIV Prevention Forum (BHPF) on 18^th^ and 19^th^ November, 2013 in Abuja, Nigeria [1]. The forum was the first of a series of conferences on biomedical HIV prevention research and development in Africa. Participants included international and regional researchers, programme managers, government officials, laboratory scientists, policy makers, and stakeholders who all shared information and ideas.

The BHPF created a platform for sharing different perspectives and experiences in HIV prevention, and provided opportunities for networking that enabled key players in the HIV prevention field in Africa to be more effective in their efforts to halt the epidemic. The BHPF also served as a platform for forging new connections across the interdependent fields of HIV programming (prevention, treatment, care and support); policy and advocacy; human rights; by linking researchers, care providers, policy makers, advocates, and people living with and affected by HIV. The ultimate goal of the linkages facilitated by the forum was to promote synergy between biomedical HIV prevention science, HIV programming, and civil society engagement. By highlighting both successes and gaps in the contribution of biomedical research to integrated country-led HIV prevention responses, the BHPF helped articulate a new agenda for biomedical HIV prevention research, including new areas of research and intervention, policy implications and evaluation, and civil society-led advocacy.

## Discussion

The BHPF hosted 342 participants from South Africa, Kenya, the United States, and Australia, with most participants (94.2%) from Nigeria. Four daily sessions included expert presentations at plenaries, roundtables and symposia. Topics discussed included the use of antiretrovirals for HIV prevention, considerations for biomedical HIV prevention among key populations (men who have sex with men (MSM), female sex workers (FSW), people who inject drugs (PWID), sero-discordant couples, young women); HIV vaccine development; HIV cure and prospect for ending the HIV epidemic; community and civil society engagement in biomedical HIV prevention; and ethical considerations for the design and implementation of biomedical HIV prevention research. Each session included opportunities for dialogue in which participants shared insight from their own knowledge and experience with the issues discussed.

A short powerpoint summary of the BHPF has been disseminated through existing national and international networks of the forum organisers, and is available from the New HIV Vaccine and Microbicide Advocacy Society. The current article provides specific details, and summarizes the specific changes or action steps identified by participants to enhance the potential impact of biomedical HIV prevention research in halting the epidemic. These action steps targeted all stakeholders, including governments, programmers, researchers and community and civil society advocates.

### Dialogue overview

The two-day forum began with discussions on systems that support biomedical HIV prevention research. Presenters gave an overview of past and present global efforts, and how to harness the potential of these tools to prevent new HIV infections, with a focus on Africa. There were discussions on the potential impact of antiretrovirals for treatment of people living with HIV, how to make eliminating mother to child transmission of HIV a priority and reality in Africa; building infrastructure to support ethical conduct of biomedical HIV prevention research in Africa; and the role of various players- such as civil society organisations, community, academia and institutional review boards (IRBs)- in the design and implementation of biomedical HIV prevention research in Africa.

The second day of the conference allowed for more in-depth discussion on the specific challenges associated with engagement of populations in HIV prevention research projects. This included presentations and discussions on engagement of young women, MSM, FSWs, and PWID. The following is a summary of the discussions and priorities of the different groups which attended the conference.

### Government leaders and programme implementers

#### Implementation of the Abuja +12 Declaration

Made in July 2013, when Heads of State of African Union countries met to review efforts made towards achieving the 2001 Abuja Declaration, the Abuja +12 Declaration [2] articulates need for an African Centre for Disease Control and Prevention to *conduct … life saving research for priority health problems in Africa and to serve as a platform to share knowledge and build capacity in responding to the public health emergencies and threats.* The Abuja +12 Declaration includes commitments to: accelerate the implementation of the 2001 Abuja Declaration [3]; review relevant laws and policies at the national and regional levels to strengthen rights based protection for all vulnerable and key populations; increase access of young people to evidence-based combination prevention programmes; promote the integration of HIV/AIDS services with sexual and reproductive health services and tuberculosis interventions; accelerate child and adolescent access to HIV treatment; and intensify research on strengthening preventive measures among others.

#### Local investment in prevention research

There has been no comprehensive accounting of African governments’ investment in biomedical HIV prevention research to date. However, with the exception of the South African government, this investment has been negligible. The US government remains the largest public-sector investor in HIV prevention research in Africa, followed by the European public sector [4]. Without significant financial participation from African governments, the research agenda risks focusing on donor governments’ priorities rather than local needs.

Consensus from the meeting was that governments in Africa need to think strategically about how to support biomedical research and development in ways that are evidence-driven, cost-effective, and reflect local needs and priorities. This would require investment in academic and industrial research and development processes including information technology (IT) and biotechnology research, development of innovation clusters and state-of-the-art core laboratories, tax incentives for research and development, support of infrastructure development and payment of competitive salaries. A national research agenda should be relevant and feasible based on local data, and supported by policies that promote the ethical conduct of research by setting standards and guidelines, providing training and support for ethics review committees, and supporting civil society watchdog organizations [5].

Plans that promote specific biomedical interventions, such as National HIV Vaccine Plans (NHVP), are less effective than broader plans that seek to strengthen a country’s capacity to contribute to research on a variety of biomedical interventions and related programmes. The NHVPs developed in 11 African countries have provided clear rules on regulatory approval, scientific and ethical reviews, biosafety and monitoring guidelines, and identified gaps to be addressed- all of which could be expanded to promote a broader biomedical prevention research agenda. Such plans should take into account lessons learned from the challenges to implementation of NHVPs in Africa, including unrealistic expectations and reliance on external organizations such as the World Health Organisation for funding [6].

### Community and civil society stakeholders

#### Build stronger partnerships and seek conversations with diverse stakeholders beyond trial communities

Until relatively recently, the understanding of “community involvement” in HIV prevention research in Africa has, with a few notable exceptions, tended to focus on the immediate local communities where the trials are taking place. Lessons learned from successful projects as well as failures [7,8], including the cancelled PrEP trials of 2005[9-12] demonstrate that a broader definition of “community stakeholders” is required to maximize the benefits of biomedical prevention research. Prevention advocacy needs to engage with national and regional media and opinion leaders, policy makers and programmers, as well as community organizations dealing with related issues beyond HIV prevention. Seeking synergy with partners working on HIV treatment and access is crucial in light of the growing body of evidence on the use of antiretrovirals for HIV prevention, such as TasP [13], and PrEP [14-17]. Similarly, the emerging though inconclusive information about a possible connection between hormonal contraceptive use and HIV risk requires engagement between advocates and programmers in the often separate fields of HIV prevention and family planning [18,19].

Given the evidence of how gender issues affect HIV risk, treatment-seeking and treatment adherence, it is crucial that biomedical prevention advocates seek collaboration with experts and organizations focused on gender and women’s empowerment. Likewise, the benefit of biomedical HIV prevention interventions for key populations- MSM, FSW, PWID - depends on effective policies that reduce stigma, enhance access to services, strengthen economic opportunities, and protect rights [20-22]. This will require collaboration across sectors that serve and advocate for these communities [23-25].

#### Develop a coherent advocacy agenda across biomedical prevention research

A prevention paradigm that includes multiple methods and indicators promotes effective synergies- for research, for policy and for advocacy. Strategies and activities need to be defined that cut across existing silos of activities and promote a common action agenda. This agenda would include advocacy for issues that affect all aspects of HIV prevention research, such as: the definition of standards of ethical practices, particularly ones supportive of research among key populations in dire need of new HIV prevention tools; ethical processes for including young adolescents in clinical research so that they can potentially benefit from the resulting data, accountability of national governments to commitments they have made to fund health, HIV prevention and treatment, and biomedical research [5,26].

#### Enhance community “research literacy.”

Community leaders and advocates also need to learn the subtle redefined vocabulary for describing HIV prevention trial processes and results to the community at large- a vocabulary that is constantly evolving. Such knowledge would contribute to meaningful engagement of community members in research processes and facilitate the development of community champions for HIV prevention research. Civil society organizations need to drive and strengthen “research literacy” and engage with researchers, institutions, and communities to enhance dialogue and understanding of prevention research, and research ethics [24].

### Researchers, institutions, and trial sponsors

#### Establish a process for determining the appropriate incentives for trial participants

The lack of guidance and consistency for determining appropriate incentives that compensate trial participants for their time and effort spent on HIV prevention research without it representing undue inducement and jeopardizing voluntary consent is a real dilemma in the HIV prevention field. Currently, most research projects do not report on these incentives and so conducting an analytical study on what may be appropriate based on study reports is difficult [27]. The field needs to look at creating a database on incentives used in different trials, settings, populations and diseases, to which research stakeholders can refer as they discuss, adapt and create incentive parameters for future studies [27].

### All stakeholders

#### Engage with the larger research field

The impact of biomedical intervention on the epidemic is inextricably linked to the context and reality of lives of individuals and communities. It is important to engage with the larger science, social science and other research fields beyond the community of stakeholders directly engaged with biomedical HIV prevention research and development, in order to recognize and actively address the social, economic, structural and cultural factors that impact risk and behavior. The importance of combining strategies that address the behavioural, biomedical and structural needs of individuals at risk of HIV prevention cannot be overemphasized. Successful national and global HIV responses need to move beyond the current efforts to medicalize HIV prevention. Rather, biomedical strategies must be well-situated within the context of the realities of the daily lives of all stakeholders [28]. Such collective efforts should be directed at seeking synergies and efficiencies between concurrent, mutually reinforcing research, policies, and programmes [29].

#### Conduct research that reflects the realities of people’s lives

Research in-turn needs to address the reality of the HIV experience and government response to the needs of its citizens. Research must be implemented in ways that build on lessons learned [30,31]. One of the realities is that people’s lives are not situated in silos. Oftentimes a FSW, or an MSM is also a drug user, and could be a person living with HIV [22,23]. Responses therefore need to be dynamic and flexible enough to adapt to the realities of the daily lives of people affected by HIV.

#### Encourage and expand private sector contributions to the national HIV/AIDS response

Such investment could include financial, skills, competencies and other resources that contribute to the collective response. So far, many national responses in Africa have done little to effectively engage the local private sector. Reliance on the donor community for the current and future HIV response is not sustainable. Government ownership of national responses is a key part of the advocacy agenda moving into the next decade [32].

#### Make cost-effective use of existing resources

New biomedical HIV prevention tools are expected to complement the existing armamentarium for the HIV response. As efforts are invested in the development of new tools, efforts also need to be invested in learning how to do increase access and utilization of existing tools. One way is to collectively learn, understand and repair the “leaks”- social, behavioral, and health system-related - along the “pipeline” from undiagnosed HIV infection to effective treatment. This way HCT, HIV treatment and PMTCT coverage can be improved [33,34]. The conduct of implementation research is one way to go. The lessons we learn now should also help to increase access to new tools, techniques and interventions for HIV prevention as they are developed in the future [31].

#### Enhance community ownership of the HIV response

Institutionalize arrangements that strengthen community ownership and contribution to care and support through local institutions and state ownership of HIV/AIDS response is also needed. Government leadership is critical, but so also must national academia, communities and end product users feel and have a sense of ownership of successes and failures in the field [25,35]. An increased sense of ownership will help facilitate roll-out of proven biomedical interventions as well as lay the groundwork for further research into HIV vaccines and even an HIV cure [31,36,37] which should indeed help “get to zero” and eventually end the global HIV epidemic: a common goal for those involved in the global HIV enterprise.

## Conclusion

The implementation of biomedical HIV prevention research has engaged African stakeholders for decades. However, there has been very few opportunities and platforms created to enable regional stakeholders to engage with one another and discuss the required concrete concerted actions to facilitate active ownership of the implementation of HIV research on the continent. The 2013 BHPF held in Abuja, Nigeria in November 2013 made important headway in identifying the key gaps, needs, opportunities and next steps for action in the field of biomedical HIV prevention research in Africa. The forum also created a platform that allowed for networking of stakeholders in the field enabling them to share resources, best practices, challenges and experiences. By highlighting key considerations for biomedical HIV prevention research and development in Africa, the forum has helped identify key advocacy issues that Civil Society can expend efforts to strengthen support for future biomedical HIV prevention research in the field on the continent.

## Competing interests

MOF, RB and BB were actively engaged with the planning and implementation of the 2013 Biomedical HIV Prevention Forum.

## Authors’ contributions

BB conceived the idea of the paper. MOF, MG, BB and RB all made substantial contributions to conception, design, acquisition, analysis and interpretation of data for this study; were involved in drafting and revising the manuscript for important intellectual content; and have given final approval of the version to be published

## List of abbreviations

AIDS: Acquired Immuno Deficiency Syndrome; BHPF: Biomedical HIV Prevention Forum; CSO: Civil Society Organisations; FSW: Female Sex Worker; HIV: Human immunodeficiency virus; IRB: Institutional Review Board; MSM: Men who have sex with Men; PrEP: Pre Exposure Prophylaxis; PWID: People Who Inject Drugs; SRH: Sexual and Reproductive Health; TB: Tuberculosis

## Declarations

This article has been published as part of BIARI, Brown University, USA and the New HIV Vaccine and Microbicide Advocacy Society. *BMC Proceedings* Volume 8 Supplement 3, 2014: 2013 Biomedical HIV Prevention Forum: Meeting Report. The full contents of the supplement are available online at http://www.biomedcentral.com/1753-6561/8/S3.

## References

[B1] 2013 Biomedical HIV prevention forum 2013 http://www.nhvmas-ng.org/forum/Accessed 12th February, 2014

[B2] Abuja +12 declaration http://abujaplus12.org/abuja12-summit-declaration/Accessed 3rd March, 2014

[B3] Abuja declaration on HIV/AIDS, tuberculosis and other related infectious diseases The African summit on HIV/AIDS, tuberculosis and other related infectious diseases Abuja, Nigeria. 24-27 April 2001. OAU/SPS/Abuja/13 http://www.un.org/ga/aids/pdf/abuja_declaration.pdf Accessed 12th February, 2014

[B4] HIV Vaccines and Microbicides Resource Tracking Working Group From Research to Reality: Investing in HIV Prevention Research in a Challenging Landscape 2013 http://www.hivresourcetracking.org/sites/default/files/Research.to_.Reality.2013.pdf Accessed 12th February, 2014

[B5] Oyedeji KS Sustaining ethical practices in HIV prevention research in Africa Presentation at the 2013 Biomedical HIV Prevention Forum, 18th - 19th November, 2013 http://nhvmas-ng.org/forum/slides/Sustaining%20ethical%20practices%20in%20HIV%20prevention%20research%20in%20Africa-Kola%20Oyedeji.pdf Accessed 12th February, 2013

[B6] Nweneka CV Implementing the 2012 Nigeria National HIV Vaccine plan Presentation at the 2013 Biomedical HIV Prevention Forum, 18th -19th November, 2013 http://nhvmas-ng.org/forum/slides/Implementing%20the%202012%20Nigeria%20National%20HIV%20Vaccine%20Plan-Chidi%20Nweneka.pdf Accessed 12th February, 2013

[B7] Van Damme L Corneli A Ahmed K Agot K Lombaard J Kapiga S Pre-exposure Prophylaxis for HIV Infection among African Women New Engl J Med 2012 367 411 22 10.1056/NEJMoa1202614 22784040 PMC3687217

[B8] Marrazzo J Ramjee G Nair G Palanee T Mkhize B Palanee T Mkhize B Nakabiito C Pre-exposure Prophylaxis for HIV in Women: Daily Oral Tenofovir, Oral Tenofovir/Emtricitabine, or Vaginal Tenofovir Gel in the VOICE Study (MTN 003) 20th Conference on Retroviruses and Opportunistic Infections, Atlanta, GA, March 3–6, 26LB. 2013

[B9] McGrory E Irvin A Heise L Research Rashomon, Lessons From the Cameroon pre-Exposure Prophylaxis Trial Site Global Campaign for Microbicides 2009 http://www.global-campaign.org/clientfiles/Cameroon.pdf (accessed February 21 2014)

[B10] Forbes A Mudaliar S Preventing Prevention Trial Failures, A Case Study and Lessons for Future Trials From the 2004 Tenofovir Trial in Cambodia Global Campaign for Microbicides 2009 http://www.global-campaign.org/clientfiles/Cambodia.pdf (accessed February 21 2014)

[B11] Ukpong M Peterson K Oral Tenofovir Controversy II: Voices From the Field, a Series of Reports of the Oral Tenofovir Trials From the Perspectives of Active Community Voices Engaged on the Field in Cambodia, Cameroon, Nigeria, Thailand and Malawi 2009 New HIV Vaccine and Microbicide Advocacy Society (NHVMAS) http://www.nhvmas-ng.org/publication/TDF2.pdf (accessed February 21 2014)

[B12] Singh JA Mills EJ The abandoned trials of pre-exposure prophylaxis for HIV: what went wrong? PLoS Med 2005 2 e234 10.1371/journal.pmed.0020234 16008507 PMC1176237

[B13] Cohen MS Chen YQ McCauley M Gamble T Hosseinipour MC Kumarasamy N Prevention of HIV-1 Infection with Early Antiretroviral Therapy New England Journal of Medicine 2011 365 6 493 505 10.1056/NEJMoa1105243 21767103 PMC3200068

[B14] Grant RM Lama JR Anderson PL McMahan V Liu AY Vargas L Preexposure chemoprophylaxis for HIV Prevention in Men Who Have Sex with Men New England Journal of Medicine 2010 363 27 2587 99 10.1056/NEJMoa1011205 21091279 PMC3079639

[B15] Baeten JM Donnell D Ndase P Mugo NR Campbell JD Wangisi J Antiretroviral Prophylaxis for HIV Prevention in Heterosexual Men and Women New England Journal of Medicine 2012 367 5 399 410 10.1056/NEJMoa1108524 22784037 PMC3770474

[B16] Thigpen MC Kebaabetswe PM Paxton LA Smith DK Rose CE Segolodi TM Antiretroviral Pre-exposure Prophylaxis for Heterosexual HIV Transmission in Botswana New England Journal of Medicine 2012 367 5 423 34 10.1056/NEJMoa1110711 22784038

[B17] Choopanya K Martin M Suntharasamai Sangkum U Mock PA Leethochawalit M Bangkok Tenofovir Study Group Antiretroviral prophylaxis for HIV infection in injecting drug users in Bangkok, Thailand (the Bangkok Tenofovir Study): a randomised, double-blind, placebo controlled phase 3 trial Lancet 2013 381 9883 2083 90 10.1016/S0140-6736(13)61127-7 23769234

[B18] Heffron R Donnell D Rees H Celum C Mugo N Were E Partners in Prevention HSV/HIV Transmission Study Team. Use of hormonal contraceptives and risk of HIV-1 transmission: a prospective cohort study Lancet Infect Dis 2012 12 1 19 26 10.1016/S1473-3099(11)70247-X 21975269 PMC3266951

[B19] Stringer EM Giganti M Carter R El-Sadr W Abrams E Stringer JS Hormonal contraception and disease progression: a multicountry cohort analysis of the MTCT-Plus Initiative AIDS 2009 23 S69 S77 10.1097/01.aids.0000363779.65827.e0 20081390 PMC3865610

[B20] Akindipe T From addiction to infection. Drug users in the world of HIV Presentation at the 2013 Biomedical HIV Prevention Forum, 18th -19th November, 2013 http://nhvmas-ng.org/forum/slides/From%20Addiction%20to%20Infection__Drug%20users%20in%20the%20world%20of%20HIV%20%20-%20Taiwo%20Akindipe.pdf Accessed 12th February, 2014

[B21] Syvertsen J HIV prevention among female sex workers: global lessons Presentation at the 2013 Biomedical HIV Prevention Forum, 18th -19th November, 2013 http://nhvmas-ng.org/forum/slides/HIV%20prevention%20among%20female%20sex%20workers__%20Global%20lessons%20-%20Jennifer%20Syvertsen.pdf Accessed 12th February, 2014

[B22] Scheibe A Money, Power and HIV: Economic influences and HIV among men who have sex with men, sex workers and people who inject drugs in sub-Saharan Africa Presentation at the 2013 Biomedical HIV Prevention Forum, 18th -19th November, 2013 http://nhvmas-ng.org/forum/slides/Money,%20Power%20and%20HIV-%20Andrew%20Scheibe.pdf Accessed 12th February, 2014

[B23] Emmanuel G The challenges of child and adolescent engagement in sexual and reproductive health research Presentation at the 2013 Biomedical HIV Prevention Forum, 18th -19th November, 2013 http://nhvmas-ng.org/forum/slides/The%20challenges%20of%20child%20and%20adolescent%20engagement%20in%20SRH%20research%20-%20Emmanuel%20Godwin.pdf Accessed 12th February, 2014

[B24] Chatani-Gada M Advocacy for action -the critical role of community partnership in HIV Prevention research Presentation at the 2013 Biomedical HIV Prevention Forum, 18th -19th November, 2013 http://nhvmas-ng.org/forum/slides/Advocacy%20for%20action,the%20critical%20role%20of%20community%20partnership%20in%20HIV%20Prevention%20research-%20Manju%20Chatani.pdf Accessed 12th February, 2014

[B25] Haire B Standard, standard, standard: who defines standards? Presentation at the 2013 Biomedical HIV Prevention Forum, 18th -19th November, 2013 http://nhvmas-ng.org/forum/slides/Standards,%20standards,%20standards__%20Who%20defines%20standards_-Bridget%20Haire.pdf Accessed 12th of February, 2014

[B26] Haire B What is the role of community advocates? Presentation at the 2013 Biomedical HIV Prevention Forum, 18th -19th November, 2013 http://nhvmas-ng.org/forum/slides/What%20is%20the%20role%20of%20community%20advocates_%20-%20Bridget%20Haire.pdf Accessed 12th of February, 2014

[B27] Brown B Ethical consideration in handling HIV prevention research protocols Presentation at the 2013 Biomedical HIV Prevention Forum, 18th -19th November, 2013 http://nhvmas-ng.org/forum/slides/Ethical%20considerations%20in%20handling%20HIV%20prevention%20research%20protocols%20-%20Brandon%20Brown.pdf Accessed 12th of February, 2014

[B28] Bukusi E Medicalisation of HIV and the Africa response Presentation at the 2013 Biomedical HIV Prevention Forum, 18th -19th November, 2013 http://nhvmas-ng.org/forum/slides/Medicalization%20of%20HIV%20and%20the%20Africa%20response%20-Elizabeth%20Bukusi.pdf Accessed 12th of February, 2014

[B29] Nwanyanwu O Getting to zero the biomedical way Presentation at the 2013 Biomedical HIV Prevention Forum, 18th -19th November, 2013 http://nhvmas-ng.org/forum/slides/Getting%20to%20Zero%20the%20Biomedical%20Way%20-%20Okey%20Nwanyanwu.pdf Accessed 12th of February, 2014

[B30] Karim SSA Antiretrovirals for HIV prevention: new hope and opportunity Presentation at the 2013 Biomedical HIV Prevention Forum, 18th -19th November, 2013 http://nhvmas-ng.org/forum/slides/Antiretrovirals%20for%20HIV%20prevention__New%20hope%20and%20opportunity%20-%20Salim%20Abdool%20Karim.pdf Accessed 12th of February, 2014

[B31] Stevens G Current options, future hopes Presentation at the 2013 Biomedical HIV Prevention Forum, 18th -19th November, 2013 http://nhvmas-ng.org/forum/slides/Current%20Options,%20Future%20Hopes-%20Gywnn%20Stevens.pdf Accessed 12th of February, 2014

[B32] Ibekwe P Leadership on finance: pace of scale up needed to realize full potential of treatment as prevention Presentation at the 2013 Biomedical HIV Prevention Forum, 18th -19th November, 2013 http://nhvmas-ng.org/forum/slides/Leadership%20on%20Finance__%20Pace%20of%20Scale%20Up%20Needed%20to%20Realize%20Full%20Potential%20of%20Treatment%20as%20Prevention%20-%20Priscilla%20Ibekwe.pdf Accessed 12th of February, 2014

[B33] Torpey K Clinical HIV treatment and its impact on community level incidence Presentation at the 2013 Biomedical HIV Prevention Forum, 18th -19th November, 2013 http://www.nhvmas-ng.org/forum/slides/Clinical%20HIV%20treatment%20and%20its%20impact%20on%20community%20level%20incidence-Kwasi%20Torpey.pdf Accessed 12th of February, 2014

[B34] Sagay AT Current state of AIDS epidemic in Nigeria: role of ARVs for PMTCT Presentation at the 2013 Biomedical HIV Prevention Forum, 18th -19th November, 2013 http://www.nhvmas-ng.org/forum/slides/Current%20State%20of%20AIDS%20Epidemic%20in%20Nigeria__%20Role%20of%20ARVs%20on%20PMTCT-%20Solomon%20Sagaay.pdf Accessed 12th of February, 2014

[B35] Fatusi A Ethical Considerations in HIV Research: Supporting role of Research & Academic Institutions Presentation at the 2013 Biomedical HIV Prevention Forum, 18th -19th November, 2013 http://www.nhvmas-ng.org/forum/slides/Ethical%20Considerations%20in%20HIV%20Research%20-%20Adesegun%20Fatusi.pdf Accessed 12th of February, 2014

[B36] Robb ML HIV vaccine field advance and reverses and a way forward Presentation at the 2013 Biomedical HIV Prevention Forum, 18th -19th November, 2013 http://nhvmas-ng.org/forum/slides/HIV%20Vaccine%20Field%20Advance%20and%20Reverses%20and%20a%20Way%20Forward%20-%20Merlin%20Robb.pdf Accessed 12th of February, 2014

[B37] Robb ML Acute HIV infection and opportunities for early intervention Presentation at the 2013 Biomedical HIV Prevention Forum, 18th -19th November, 2013 http://nhvmas-ng.org/forum/slides/Acute%20HIV%20Infection%20and%20Opportunities%20for%20Early%20Intervention%20-%20Merlin%20Robb.pdf Accessed 12th of February, 2014

